# Variation in Dehydration Tolerance, ABA Sensitivity and Related Gene Expression Patterns in D-Genome Progenitor and Synthetic Hexaploid Wheat Lines

**DOI:** 10.3390/ijms10062733

**Published:** 2009-06-18

**Authors:** Yumeto Kurahashi, Akihiro Terashima, Shigeo Takumi

**Affiliations:** Laboratory of Plant Genetics, Graduate School of Agricultural Science, Kobe University, Nada-ku, Kobe 657-8501, Japan; E-Mails: 096a512a@stu.kobe-u.ac.jp (Y.K.); at3818@yahoo.co.jp (A.T.)

**Keywords:** abscisic acid, allopolyploidization, drought stress, natural variation, synthetic wheat

## Abstract

The wild wheat *Aegilops tauschii* Coss. has extensive natural variation available for breeding of common wheat. Drought stress tolerance is closely related to abscisic acid (ABA) sensitivity. In this study, 17 synthetic hexaploid wheat lines, produced by crossing the tetraploid wheat cultivar Langdon with 17 accessions of *Ae. tauschii,* were used for comparative analysis of natural variation in drought tolerance and ABA sensitivity. *Ae. tauschii* showed wide natural variation, with weak association between the traits. Drought-sensitive accessions of *Ae. tauschii* exhibited significantly less ABA sensitivity. D-genome variations observed at the diploid genome level were not necessarily reflected in synthetic wheats. However, synthetic wheats derived from the parental *Ae. tauschii* accessions with high drought tolerance were significantly more tolerant to drought stress than those from drought-sensitive accessions. Moreover, synthetic wheats with high drought tolerance showed significantly higher ABA sensitivity than drought-sensitive synthetic lines. In the hexaploid genetic background, therefore, weak association of ABA sensitivity with drought tolerance was observed. To study differences in gene expression patterns between stress-tolerant and -sensitive lines, levels of two *Cor*/*Lea* and three transcription factor gene transcripts were compared. The more tolerant accession of *Ae. tauschii* tended to accumulate more abundant transcripts of the examined genes than the sensitive accession under stress conditions. The expression patterns in the synthetic wheats seemed to be additive for parental lines exposed to drought and ABA treatments. However, the transcript levels of transcription factor genes in the synthetic wheats did not necessarily correspond to the postulated levels based on expression in parental lines. Allopolyploidization altered the expression levels of the stress-responsive genes in synthetic wheats.

## 1. Introduction

Abiotic stress signal pathways have been studied using artificially generated mutants in the model plant *Arabidopsis thaliana*. However, naturally occurring variants still provide an important alternative source of genetic variation [1]. Recent progress in the analysis of natural variation in *Arabidopsis* demonstrates the usefulness of wild populations for identification of genetic loci accounting for naturally occurring variation in abiotic stress tolerance [2–4]. Naturally occurring variants are useful in determining which specific allelic variants exist in nature, where they might either be neutral or have a selective advantage under specific conditions [1].

Abiotic stresses such as drought, temperature and salinity reduce yield of common wheat (*Triticum aestivum* L.). Under abiotic stress conditions, plant cells undergo drastic physiological, biochemical and metabolic changes leading to the development of stress tolerance at the cellular level. One of the mechanisms behind development of stress tolerance is induction of the *Cor* (cold-responsive)/*Lea* (late-embryogenesis-abundant) gene family; COR/LEA proteins promote the development of stress tolerance by protecting cellular components from stress [5]. A number of *Cor*/*Lea* genes are not only responsive to low temperature but also to drought and abscisic acid (ABA). Many low temperature- and drought-inducible genes contain both C-repeat (CRT)/dehydration-responsive element (DRE) and ABA-responsive element (ABRE) motifs in their promoters, and these *cis* elements are considered to function independently. Expression of these *Cor*/*Lea* genes is regulated by major transcription factors in the CBF/DREB and AREB/ABF families under abiotic stress conditions such as low temperature and osmotic stress [6].

ABA is one of the key plant hormones responding to environmental stress [7]. ABA is synthesized de novo mainly in response to drought and high salinity stress in vegetative tissues [8,9]. Several *Cor*/*Lea* genes, the expression of which correlate with increased freezing and drought tolerance, are also responsive to exogenous ABA [10], and ABRE sequences exist in the promoter regions of these *Cor*/*Lea* genes in *Arabidopsis* [11–13]. The *Arabidopsis* basic region/leucine zipper (bZIP)-type AREB/ABF proteins bind as dimers to ABRE and activate ABA-dependent gene expression [14–16].

CRT/DRE, ABRE and other cold-responsive motifs have been identified in the 5′ upstream regions of many wheat *Cor*/*Lea* genes such as *Wdhn13* and *Wrab17* [17–20]. Wheat *CBF* homologs such as *TaCBF*, *TaDREB1* and *WCBF2* encode transcription factors recognizing the CRT motif [21–24]. Of the *CBF* homologs, *TaDREB1* expression is clearly responsive to drought stress and ABA. In addition, wheat bZIP-type transcription factors, WABI5, TaOBF1 and WLIP19, bind to the *Cor*/*Lea* promoter regions and directly activate *Cor*/*Lea* gene expression [25,26]. Expression of *WABI5*, *TaOBF1* and *WLIP19* is increased after drought stress and ABA treatment in leaves of common wheat seedlings. Thus, *Cor*/*Lea* gene expression mediated by CBF/DREB and bZIP-type transcription factors might play a central role in development of abiotic stress tolerance in wheat.

*Aegilops tauschii* Coss. (syn. *Ae. squarrosa* Eig.), a wild relative of wheat, is one of the diploid progenitors of common wheat [27,28]. Common wheat is an allohexaploid species that originated from natural hybridization between tetraploid wheat (*Triticum turgidum* L.), including emmer and durum wheats, and *Ae. tauschii* [29,30]. *Ae. tauschii* is widely distributed in Eurasia and shows abundant genetic variation [31–36]. The birthplace of common wheat is considered to lie within the area comprising Transcaucasia and the southern coastal region of the Caspian Sea [37]. Therefore, the *Ae. tauschii* populations involved in the origin of common wheat are limited to a narrow distribution range relative to the entire species range, suggesting that this species holds vast genetic diversity that is not represented in common wheat [37]. Natural variation in the *Ae. tauschii* population provides potential for improving modern varieties of common wheat. In fact, hexaploid synthetic wheat derived from crosses between tetraploid wheat and *Ae. tauschii* has the potential to provide new genetic variation for abiotic stress tolerance [38].

In the present study, we surveyed naturally occurring genetic variation in drought tolerance and ABA sensitivity in *Ae. tauschii*. Next, we examined expression of the natural variation in synthetic hexaploid lines produced by crossing the tetraploid wheat cultivar Langdon with *Ae. tauschii* accessions. In addition, we compared expression of the *Cor*/*Lea* and *Cor*/*Lea* transcription factor genes to analyze the molecular nature of the natural variation in both synthetic wheats and their parental accessions. Based on these results, we discuss the relationship between drought tolerance and ABA sensitivity and the usefulness of *Ae. tauschii* variation in wheat breeding.

## 2. Results

### 2.1. Natural Variation in Dehydration Tolerance and ABA Sensitivity in Aegilops Tauschii

**Figure 1.**
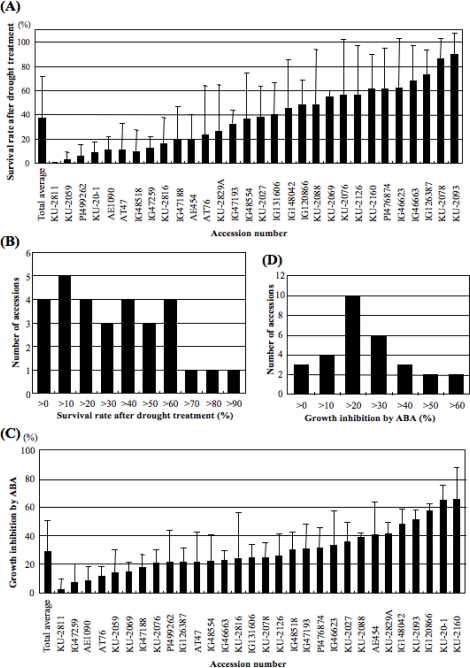
Natural variation in drought tolerance and ABA sensitivity in 30 *Ae. tauschii* accessions. (A) Survival rates (%) after 24 h drought stress. Means ± SDs were calculated from data from three independent experiments. In each experiment, at least 10 plants were tested. (B) Frequency distribution of the drought tolerance levels (%) in the 30 *Ae. tauschii* accessions. (C) Growth inhibition rate (%) in the presence of 10 μM ABA. Means ± SD were calculated from data from three independent experiments. In each experiment, at least five plants were tested. (D) Frequency distribution of ABA sensitivity in the 30 *Ae. tauschii* accessions.

Thirty accessions of *Ae. tauschii* were examined to evaluate the level of drought tolerance and exogenous ABA sensitivity (Table 1). The drought tolerance level was calculated based on the survival rate of 10-d-old seedlings after 24 h of drought treatment at 25°C; it varied widely from 0 ± 0.0% (KU-2811) to 90.0 ± 17.3% (KU-2093) in the 30 *Ae. tauschii* accessions (Figure 1A). All accessions from Iran (n=7) showed high tolerance to drought stress, whereas Transcaucasus accessions exhibited comparatively low levels of drought tolerance. The average survival was 37.4 ± 34.1%. Thirteen of all the accessions examined showed less than 30% survival, and only three accessions exhibited more than 70% survival under drought conditions (Figure 1B).

It was previously reported that cultivar difference of responsiveness to exogenous ABA was corresponding to the difference of abiotic stress tolerance in common wheat [19]. Here, we called the responsiveness to exogenous ABA as ABA sensitivity. ABA sensitivity was evaluated by inhibition of post-germination growth in the presence of 10 μM ABA. ABA greatly reduced root length in most accessions of *Ae. tauschii*. The ABA sensitivity was measured by the relative growth inhibition rate (% reduction in the presence ABA relative to growth in the absence of ABA).

It also varied widely from 2.0 ± 7.3% (KU-2811) to 65.4 ± 22.4% (KU-2160) in the 30 *Ae. tauschii* accessions (Figure. 1C, 1D). The average ABA sensitivity was 29.1 ± 21.1%.

**Figure 2.**
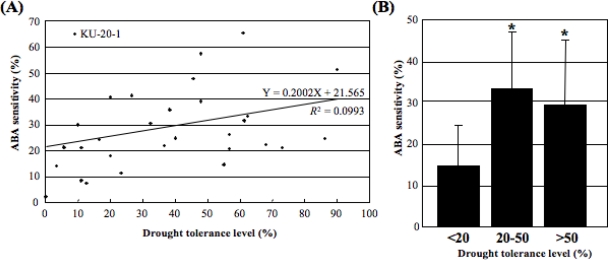
Correlation between drought tolerance and ABA sensitivity in *Ae. tauschii*. (A) Scatter plot showing drought tolerance and ABA sensitivity in 30 *Ae. tauschii* accessions. (B) Comparison of ABA sensitivity for three *Ae. tauschii* groups (excluding KU-20-1, which had a distinct level of drought tolerance). Student’s *t*-test was used to test for statistical significance (*P < 0.05) between the different categories of drought tolerance.

The ABA sensitivity of each of the 30 *Ae. tauschii* accessions was compared with its drought tolerance. KU-20-1 showed exceptionally low drought tolerance in spite of high ABA sensitivity. In the 29 other accessions, the correlation between drought tolerance and ABA sensitivity was positive (*R^2^*=0.0993), but not significant (Figure 2A). These 29 accessions were classified into three groups: drought-sensitive (<20% survival rate), moderately tolerant (20–50%) and highly tolerant (>50%) groups. Student’s *t*-test showed that the ABA sensitivity of the drought-sensitive group was significantly (*P* < 0.05) lower than that of the moderately and highly tolerant groups (Figure 2B).

### 2.2. Expression of Dehydartion Tolerance and ABA Sensitivity in Synthetic Wheats

Seventeen lines of synthetic wheats were used to evaluate the level of drought tolerance and ABA sensitivity. These 17 lines were derived from interspecific crosses between a tetraploid wheat accession *Triticum durum* cv. Langdon and 17 *Ae. tauschii* accessions (Table 1). The somatic chromosome number was 42 in all F_3_ seeds examined from single F_2_ plants of each synthetic. All the synthetics were highly fertile and there was no segregation of plants with excessively reduced height (indicative of haploidy or aneuploidy) in the F_2_ populations (data not shown), suggesting that the synthetics were hexaploid and stable.

**Figure 3.**
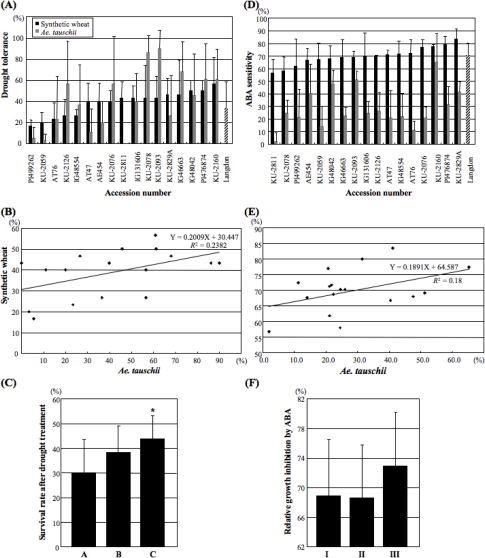
Variation in drought tolerance and ABA sensitivity of 17 synthetic wheats and their parental lines. (A) Drought tolerance revealed by survival rate after a 4 d drought treatment. (B) Scatter plot of drought tolerance in the synthetics and parental *Ae. tauschii* accessions. (C) Comparison of drought tolerance for three categories of synthetics having parental *Ae. tauschi* lines with distinct levels of drought tolerance. Student’s *t*-test was used to test for statistical significance (*P < 0.05) compared with the drought-sensitive group with low drought tolerance. A, drought-sensitive accessions; B, accessions with moderate drought tolerance; C, highly drought tolerant accessions. (D) ABA sensitivity based on relative growth inhibition (%) due to 20 μM ABA treatment. Means ± SD were calculated from data from three independent experiments. In each experiment, at least five plants were tested. (E) Scatter plot of ABA sensitivity in the synthetics and parental *Ae. tauschii* accessions. (F) Comparison of ABA sensitivity for three categories of synthetics having parental *Ae. tauschi* lines with distinct levels of ABA sensitivity. I, low ABA-sensitivity accessions; II, moderately ABA-sensitive accessions; III, highly ABA-sensitive accessions.

Seedlings of the synthetic wheat lines were treated by dehydration stress for four days, because the synthetic wheats were tolerant to the 24-h drought treatment. The synthetic wheats showed wide variation for both drought tolerance and sensitivity to exogenous ABA, but the range of variation in the synthetic wheats was narrower than in the parental *Ae. tauschii* accessions (Figures 3A, 3D). The average drought tolerance level was 38.3 ± 11.3% in synthetic wheats, and the tolerance levels varied from 16.7 ± 5.8% (Langdon x PI499262) to 56.7 ± 25.2% (Langdon x KU-2160). There was a positive correlation of drought tolerance levels between synthetic wheats and the parental *Ae. tauschii* accessions (*R^2^*=0.238), but the correlation was not significant (*P*=0.24) (Figure 3B). The 17 synthetic lines were divided into three groups based on the level of drought tolerance of their parental *Ae. tauschii* accessions. Group A consisted of four lines, of which the parental *Ae. tauschii* accessions PI499262, KU-2059, KU-2811 and AT47 showed drought sensitivity (less than 15% survival rates). A total of six synthetic wheats were classified into group B and seven into group C, for which the respective parental *Ae. tauschii* accessions exhibited moderate (20–50%) and high (>50%) drought tolerance levels. The group C lines showed significantly higher drought tolerance than group A (Figure 3C). In group C, the synthetic lines were more tolerant to drought stress than the parental Langdon (Figure 3A).

The average ABA sensitivity was 70.3 ± 6.9% in synthetic wheats, and varied from 56.7 ± 10.6% (Langdon x KU-2811) to 83.5 ± 8.2% (Langdon x KU-2829A) under the 20 μM ABA condition. The correlation (*R*=0.424) of the ABA sensitivity between synthetic wheats and parental *Ae. tauschii* accessions was not significant (*P*=0.09) (Figure 3E). The 17 synthetic wheats were classified into three groups based on the ABA sensitivities of their parental *Ae. tauschii* accessions. Five synthetic lines with less than 21% of their parental *Ae. tauschii* sensitivity belonged to group I and seven with 21–40% sensitivity belonged to group II. Group III consisted of five accessions with more than 40% the ABA sensitivity of their parental *Ae. tauschii* accessions. No significant difference, however, was observed among the three groups. Although all parental *Ae. tauschii* accessions showed lower ABA sensitivity than Langdon, seven synthetic wheat lines were more sensitive to exogenous ABA than the parental Langdon (Figure 3D).

**Figure 4.**
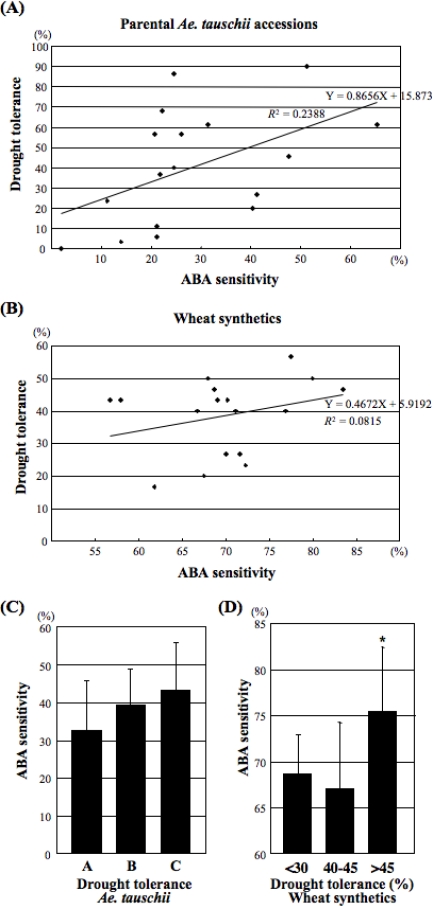
Correlation between drought tolerance and ABA sensitivity in synthetic wheats and parental accessions. (A) Scatter plot of drought tolerance and ABA sensitivity in the parental *Ae. tauschii* accessions. (B) Scatter plot of drought tolerance and ABA sensitivity in the synthetic lines. (C) Comparison of ABA sensitivity for three groups of *Ae. tauschii* accessions with distinct levels of drought tolerance. A, drought-sensitive accessions; B, accessions with moderate drought tolerance; C, highly drought-tolerant group. (D) Comparison of ABA sensitivity for three groups of synthetic wheats with distinct levels of drought tolerance. Student’s *t*-test was used to test for statistical significance (*P < 0.05) compared with the >45% survival group with low drought tolerance.

The relationship between the level of drought tolerance level and ABA sensitivity was studied in the hexaploid genetic background. Correlation (*R*) values between drought tolerance and ABA sensitivity were 0.49 (*P*=0.122) in the parental *Ae. tauschii* accessions and 0.29 (*P*=0.26) in the synthetic wheats (Figures 4A, 4B). No significance was observed between the drought tolerance level and ABA sensitivity at either the diploid or hexaploid level. The range of variation in the level of drought tolerance and ABA sensitivity was narrower in the synthetic lines than in the parental *Ae. tauschii* accessions. The ABA sensitivity of the 17 synthetic wheat lines was also compared with the level of drought tolerance of both parental *Ae. tauschii* accessions and synthetic lines. No significant difference in ABA sensitivity was observed among the three groups based on the drought tolerance of the parental *Ae. tauschii* accessions (Figure 4C). The highly drought-tolerant group with more than 45% tolerance in the synthetic wheats was significantly more sensitive to exogenous ABA treatment than the other groups (Figure 4D).

### 2.3. Expression Patterns of *Cor*/*Lea* Genes under Stress Conditions

To compare gene expression patterns between lines with low and high ABA sensitivity, two *Ae. tauschii* accessions, KU-2811 (low ABA sensitivity) and KU-2829A (high ABA sensitivity), and synthetic lines derived from them were selected. Both KU-2811 and a synthetic wheat line derived from Langdon and KU-2811 showed the lowest ABA sensitivity, while KU-2811 was the most drought-sensitive. The ABA sensitivity of both KU-2829A and a synthetic wheat line derived from Langdon and KU-2829A was high, and the drought tolerance of the KU-2829A-derived synthetic line was also high.

**Figure 5.**
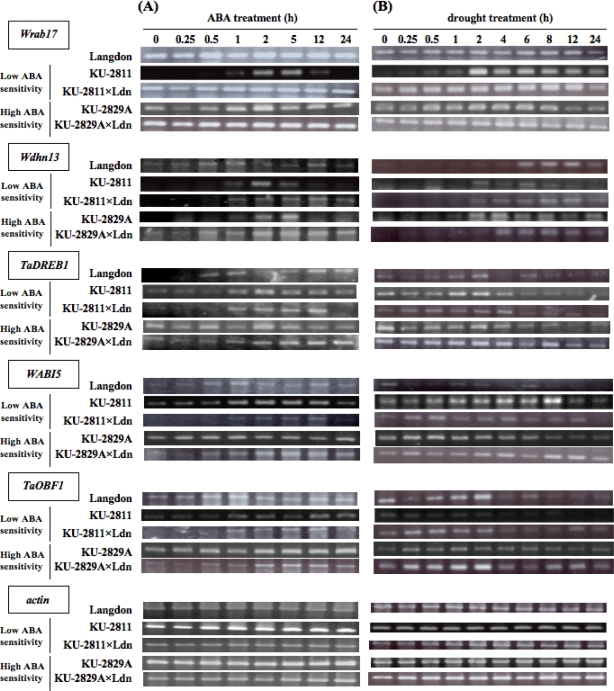
Expression patterns of two *Cor*/*Lea* genes (*Wrab17* and *Wdhn13*) and three transcription factor genes (*TaDREB1*, *WABI5* and *TaOBF1*) in the synthetic wheats, parental *Ae. tauschii* accessions and Langdon after ABA and drought-stress treatment. (A) ABA-responsive expression. Gene expression patterns were revealed by RT-PCR analysis using the same set of RNA preparations. *Actin* was used as internal control. Total RNA was extracted from leaves of seedlings after the indicated times in the 20 μM ABA treatment. (B) Drought-responsive expression. Total RNA was extracted from leaves of seedlings after the indicated drought treatment.

Two *Cor*/*Lea* genes, *Wrab17* and *Wdhn13*, are responsive to exogenous ABA and drought stress treatment [17–19]. Expression levels of the *Actin* gene used as an internal control were not changed by stress treatment in any of the accessions and lines examined. *Wrab17* transcript accumulation was clearly induced by ABA and drought treatment in both *Ae. tauschii* accessions (Figure 5A). KU-2829A accumulated *Wrab17* transcripts more abundantly than KU-2811 under both stress conditions. In Langdon and the two synthetic wheats, induction of *Wrab17* expression was not clear, and the accumulation was highly abundant. No significant difference in expression pattern was observed for the ABA and drought treatments among Langdon and the two synthetic wheats.

*Wdhn13* expression was responsive to ABA and drought stress in all accessions and lines examined (Figure. 5B). The *Wdhn13* transcript levels were higher in KU-2829A than in KU-2811 under the drought stress conditions. The ABA- and drought-responsive expression patterns in both synthetics seemed to correspond to the sum of those of Langdon and the parental *Ae. tauschii* accessions. ABA-responsive accumulation of *Wdhn13* transcripts occurred earlier in the KU-2829A-derived synthetic line than in the KU-2811-derived line. Under drought stress, the *Wdhn13* transcript level reached a high plateau earlier in the KU-2829A-derived synthetic line than in the KU-2811-derived line.

### 2.4. Expression Patterns of Transcription Factor Genes under Stress Conditions

In previous studies, we reported that *Wrab17* and *Wdhn13* expression is at least partly controlled by CBF/DREB-related transcription factors as well as by WABI5, WLIP19 and TaOBF1 transcription factors [19,20,24,25]. In the present study, expression patterns of three transcription factor genes, *TaDREB1*, *WABI5* and *TaOBF1*, were compared for lines with low and very high ABA sensitivity. The RNA samples used for the analysis of expression of these transcription factor genes were the same ones used for profiling *Wrab17* and *Wdhn13* gene expression.

Expression of *TaDREB1*, *WABI5* and *TaOBF1* was responsive to exogenous ABA treatment in Langdon and the parental *Ae. tauschii* accessions (Figure 5A). KU-2829A more abundantly accumulated *TaDREB1* and *WABI5* transcripts than KU-2811 after ABA treatment. *TaDREB1* and *WABI5* transcript levels were higher in the KU-2829A-derived synthetic line than in the KU-2811-derived line. Expression of the three transcription factor genes was responsive to drought stress treatment, but the induction was more rapid than after ABA treatment (Figure 5B). The drought-responsive expression patterns of *TaDREB1*, *WABI5* and *TaOBF1* in both synthetics seemed to correspond to the sum of those of Langdon and the parental *Ae. tauschii* accessions.

**Figure 6.**
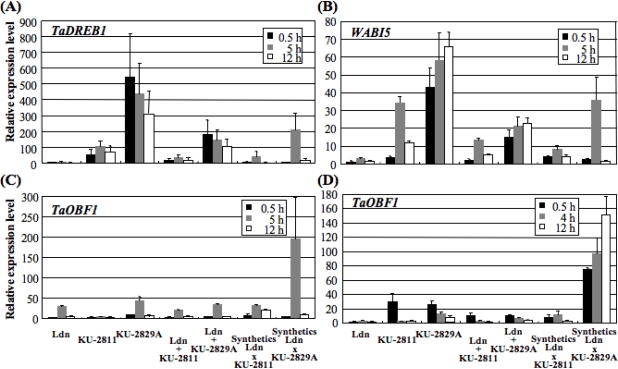
Comparison of transcript levels in synthetic wheats and their parental lines. Quantitative RT-PCR analysis was conducted using leaves from ABA- and drought stress-treated seedlings. The postulated levels in the synthetic wheats were calculated as 2:1 ratio mixtures of the transcript levels of the parental Langdon and *Ae. tauschii* accessions. Each transcript level was represented as the value relative to the Langdon level at 0.5 h. (A) *TaDREB1* transcript levels after ABA treatment. (B) *WABI5* transcript levels after ABA treatment. (C) *TaOBF1* transcript levels after ABA treatment. (D) *TaOBF1* transcript levels under drought conditions.

For quantitative comparison of transcript levels of *TaDREB1*, *WABI5* and *TaOBF1*, real-time RT-PCR analysis was conducted using RNA samples obtained after exogenous ABA (0.5, 5 and 12 h) and drought (0.5, 4 and 12 h) treatments. Transcript levels relative to Langdon (0.5 h) were calculated for both the parental *Ae. tauschii* accessions and the synthetic wheat lines. The transcript levels in the synthetic wheats were also compared with the postulated transcript levels, which were calculated as a 2:1 mixture of Langdon and the parental *Ae. tauschii* accession. Transcript levels of *TaDREB1*, *WABI5* and *TaOBF1* in KU-2829A were much higher than those in KU-2811 after ABA treatment (Figure 6). The *TaDREB1*, *WABI5* and *TaOBF1* transcript levels in the KU-2829A-derived synthetic line were also higher than those in the KU-2811-derived line, but they were different than the postulated levels. Under drought stress conditions, *TaOBF1* transcript in KU-2829A and the KU-2829A-derived synthetic line accumulated more abundantly than in KU-2811 and the KU-2811-derived synthetic line. Similarly to the expression levels after ABA treatment, the *TaOBF1* transcript levels in the synthetic lines differed from the postulated levels.

## 3. Discussion

Naturally occurring genetic variation is one of the most important basic resources for plant biology [1]. *Ae. tauschii* has a wide natural species range in central Eurasia, ranging from northern Syria and Turkey to Western China. In previous studies, the natural variation of *Ae. tauschii* populations was analyzed for several traits, including flowering and morphological traits and hybrid lethality in crosses with tetraploid wheat [33–35]. *Ae. tauschii* is also known as the D-genome progenitor of hexaploid bread wheat. Therefore, hexaploid synthetic wheat lines can be artificially produced through allopolyploidization between tetraploid wheat and *Ae. tauschii* [39,40], implying that agronomically important genes from natural variation of the *Ae. tauschii* population are available for wheat breeding by making synthetic wheats. In this study, natural variation in drought tolerance level and ABA sensitivity was evaluated in the *Ae. tauschii* population. ABA is a plant hormone known to be tightly associated with environmental stress such as drought and salt [7]. *Ae. tauschii* showed wide natural variation for the level of drought tolerance and ABA sensitivity (Figure 1), and weak association between the two traits was observed (Figure 2). Drought-sensitive accessions of *Ae. tauschii* exhibited significantly less ABA sensitivity. This observation agreed with previous knowledge of the function of ABA in abiotic stress responses. Therefore, the natural variation analysis in this study suggested that ABA plays at least an important partial role in development of drought stress tolerance in wild diploid wheat. However, the correlation between the level of drought tolerance and ABA sensitivity was low in the *Ae. tauschii* accessions. Seven accessions of *Ae. tauschii* from Iran, mostly originating from the Caspian coastal region, showed high levels of drought tolerance. Drought-sensitive accessions of *Ae. tauschii* were distributed over a wide area including Syria, the Transcaucasus, Afghanistan and China. The correlation of drought stress response with geographical origin of the accessions was not necessarily found for ABA sensitivity. The low correlation value might be due to the presence of other factors contributing to drought stress tolerance or to methodological problems used to assess the level of drought tolerance. In this study, the survival rate after drought-stress treatment was used as a measure of the drought tolerance level. Other bioassay systems should also be examined to evaluate drought tolerance.

The 17 synthetic hexaploids used in this study were derived through endoreduplication forming triploid gametes in the interspecific ABD hybrids obtained by crossing Langdon with 17 of the *Ae. tauschii* accessions. Thus, the genetic variation among the lines fundamentally originated from the D genome because the A and B genomes of the synthetics were all derived from the same genotype. All 17 lines were somatically stable with 2n = 42 and did not show any abnormal phenotype, such as hybrid weakness or hybrid lethality, often observed in triploid hybrids between tetraploid wheat and *Ae. tauschii* [33,41,42]. Therefore, these 17 synthetic wheat lines provide a large potential not only for use in wheat breeding but also in the study of transmission and expression of genetic variation in the D genome in a hexaploid genomic background. In this study, large variation was observed in the level of drought tolerance and ABA sensitivity among these synthetics. For both traits, correlation between the synthetic wheats and parental *Ae. tauschii* accessions was positive, but not statistically significant. Therefore, the D-genome variations observed at the diploid genome level were not necessarily reflected in the synthetic wheats. However, the synthetic wheats derived from the parental *Ae. tauschii* accessions with high drought tolerance were significantly more tolerant to drought stress than those from the drought-sensitive accessions (Figure 3C). Moreover, the synthetic wheats with high drought tolerance showed significantly higher ABA sensitivity than the drought-sensitive synthetic lines (Figure 4D). In the hexaploid genetic background, therefore, some association of ABA sensitivity with drought tolerance could be observed. These results indicated that *Ae. tauschii* accessions with high levels of abiotic stress tolerance are good candidates for genetic resources to breed abiotic stress-tolerant cultivars of bread wheat.

Two *Cor*/*Lea* genes, *Wrab17* and *Wdhn13*, were responsive to exogenous ABA and drought-stress treatment in *Ae. tauschii* as well as common wheat [19]. In common wheat, these genes are also cold-inducible, and the timing and level of induction are correlated with the level of freezing tolerance under low temperature in two cultivars with contrasting levels of freezing tolerance [43]. Similarly, KU-2829A, with high ABA sensitivity and drought tolerance, showed more rapid response in *Cor*/*Lea* gene expression than the ABA-insensitive and drought-sensitive KU-2811 (Figure 5). Although growth inhibition following ABA treatment was dramatic in KU-2811, for unknown reasons, *Cor*/*Lea* expression was ABA-responsive. The ABA responsiveness of KU-2811 seems to be quite low in the growth response but not completely disrupted in the gene response, suggesting that *Wrab17* and *Wdhn13* are involved in downstream genes functioning in the ABA-dependent signal pathway for development of drought tolerance. In the 5′ upstream regions of *Wrab17* and *Wdhn13*, several *cis*-elements, such as CRT/DRE and ABRE, were recognized in our previous study [20]. *TaDREB1* is a candidate for the transcription factor of *Wrab17* and *Wdhn13* [22], and *WABI5* and *TaOBF1* directly activate *Wrab17* and *Wdhn13* expression in common wheat [25,26]. The more tolerant accession KU-2829A tended to accumulate transcripts of the transcription factor genes more abundantly than the sensitive accession KU-2811 under stress conditions (Figure 6). These results indicated that the differences in abiotic stress responses between the two *Ae. tauschii* accessions might be due to further upstream genetic factor(s) in the ABA-dependent signal pathway. In *Arabidopsis*, the Versailles core collection contains significant natural variation in freezing tolerance, CBF gene sequences and *CBF* and *Cor*/*Lea* gene expression patterns [44]. Although there tends to be more *CBF* and *Cor* gene expression in freezing-tolerant accessions, neither *CBF* nor *Cor* gene expression is closely correlated with freezing tolerance, and the *CBF* genes alone cannot explain all differences in the level of freezing tolerance [44], which implies difficulties in assessing the function of single transcription factors. Major genetic loci resulting in phenotypic differences in drought tolerance among the *Ae. tauschii* accessions should be identified via a mapping-based approach.

Allopolyploidization alters gene expression profiles in allopolyploid cells [45]. Gene expression patterns are stochastically and epigenetically changed during the generation of allopolyploid plants [46–48]. Synthetic wheat lines are powerful tools to study the effects of polyploidization on gene expression [49]. For example, a significant change in the *WDREB2* gene expression pattern has been reported in hexaploid synthetic wheats [50]. Our RT-PCR analysis of *Cor*/*Lea* genes and the genes encoding their transcription factors indicated that their expression patterns in the hexaploid synthetics seemed to be additive of those in their parental lines under both exogenous ABA treatment and drought conditions (Figure 5). However, real-time RT-PCR analysis demonstrated that accumulation of transcripts of transcription factor genes in the hexaploid synthetics did not necessarily correspond to the postulated levels based on expression in parental lines (Figure 6). These observations indicated that hexaploidization through production of the synthetic wheats altered the expression levels of ABA- and drought-responsive genes under stress conditions. It was quite difficult to evaluate the effect of the altered expression levels on stress tolerance. In fact, the drought tolerance levels of the synthetic wheats were generally higher than those of their parental *Ae. tauschii* accessions (Figure 3D). Alteration of gene expression profiles through allopolyploidization should be further studied in synthetic wheats using transcriptome-based approaches.

## 4. Experimental Section

### 4.1. Plant Materials

A total of 30 *Ae. tauschii* accessions representing the entire natural habitat range of the species was used in the study (Table 1). Passport data for these accessions, including the geographical coordinates of the original collection sites, have been given in Matsuoka *et al*. [33,34]. For each accession, we used seeds propagated from a single plant by self-pollination. Twelve hexaploid synthetic wheats previously reported [33,51] were used, and five synthetic hexaploids were additionally produced in this study (Table 1). For production of the 17 synthetics, tetraploid wheat accession *Triticum durum* cv. Langdon was used as the female parent and crossed with each of the 17 *Ae. tauschii* accessions. The F_1_ progeny were grown and selfed to produce synthetics (herein designated the F_2_ generation). All 17 synthetics were independently generated through unreduced gamete formation in each of the triploid F_1_ hybrids [40]. The synthetics thus contained the A and B genomes from Langdon and the diverse D genomes originating from the *Ae. tauschii* male parents. All grew normally and showed none of the hybrid lethality or weakness, such as necrosis and chlorosis, often observed in triploid hybrids between tetraploid wheats and *Ae. tauschii* [33,41,42]. Somatic chromosome numbers were determined from root-tip mitotic preparations of three F_3_ seeds from one F_2_ plant of each synthetic, using the standard acetocarmine squash method.

### 4.2. Bioassay for Abiotic Stress Tolerance

Seedlings were grown under standard conditions (23°C) according to Kobayashi *et al.* [43]. For analysis of drought tolerance, 10-day-old seedlings (n=20) of *Ae. tauschii* were removed from the soil and kept on dry filter paper for 24 h at 25°C. Seven-day-old seedlings (n=20) of synthetic wheats (F_3_ generation) were dehydrated on dry paper for 4 d at 23°C. Stress-treated seedlings were transferred back to the standard conditions, and on the 7th day after transfer, the number of surviving seedlings was recorded. To bioassay ABA sensitivity based on post-germination growth, seeds were imbibed under tap water for 5 h and kept overnight at 4°C. Imbibed seeds were placed in a glass petri dish containing filter paper wetted with distilled water, and incubated for 24 h at 20°C in darkness. Ten synchronously germinated seeds were further treated with distilled water or 10 or 20 μM ABA solution under the same conditions as the germination assay. After 3 d, the length of primary roots was recorded. The whole experiment was repeated at least three times. The data were statistically analyzed using JMP software ver. 5.1.2 (SAS Institute). Correlations among the morphological traits were estimated based on Pearson correlation coefficient values.

### 4.3. Expression Analysis

Total RNA was extracted from the leaves of 7-d-old seedlings for various times under stress conditions. Seven-day-old seedlings were also treated with a solution containing ABA (20 μM) by a foliar spray or were dehydrated on dry filter paper in a desiccator. The transcript accumulation of *Cor*/*Lea* genes such as *Wdhn13* and *Wrab17* and their three transcription factor genes, *TaDREB1*, *WABI5* and *TaOBF1*, was detected by RT-PCR amplification as previously reported [19,20]. RT-PCR for *TaDREB1* was conducted with the following gene-specific primer pair: 5′-AGTCTCCTCCTTCTC TTATCTC-3′ and 5′-TTCTTGTACCCGTTGACTTATG-3′. The actin gene (*Actin*) was used as an internal control with the following gene-specific primer pair: 5′-GGCTGGTTTTGCTGGTGACGA AT-3′ and 5′-AATGAAGGAAGGCTGGAAGAGGA-3′. The PCR products were separated by electrophoresis through a 1.5% agarose gel and stained with ethidium bromide for detection.

Quantitative RT-PCR was performed using a Thermal Cycler Dice^®^ Real Time System (Takara-Bio, Ohtsu, Japan) and gene-specific primer sets. For *TaDREB1* and *WABI5*, the following two primer pairs were designed: 5′-TCTCTCTCGTCCCTCTTCTC-3′ and 5′-TTTTCCTCCTTCCACTTCTT-3′, and 5′-GGGATTGTGAGGGGGAGGAG-3′ and 5′-GGCGGACTCCCTGTTCTTGA-3′, respectively. For the other genes, the same primer pairs used for RT-PCR amplification were used in the quantitative RT-PCR. As an endogenous control, *Actin* was used. The rate of amplification was monitored by SYBR^®^ Premix Ex Taq^™^ II (Takara-Bio) according to the manufacturer’s protocol. Results were presented as 2^−ΔCt^, where Ct is the number of PCR cycles required to reach the log phase of amplification for the examined genes minus the number of cycles to reach the same stage for *Act*, and then were represented as values relative to the transcript levels in samples of Langdon obtained at 0.5 h.

## 5. Conclusions

A wild wheat progenitor, *Ae. tauschii*, had large genetic variation in drought tolerance and exogenous ABA sensitivity. The level of drought tolerance was at least partly related to ABA sensitivity. Similarly, synthetic wheats derived from hybrids between Langdon and *Ae. tauschii* accessions showed wide variations in drought tolerance and ABA sensitivity, although no significant correlation between the synthetic wheats and their parental *Ae. tauschii* accessions was observed. However, synthetic wheats derived from the parental *Ae. tauschii* accessions with high drought tolerance were significantly more tolerant to drought stress than those from the drought-sensitive accessions. Therefore, *Ae. tauschii* accessions with high levels of abiotic stress tolerance are expected to be useful resources to breed abiotic stress-tolerant cultivars of bread wheat. Allohexaploidization altered the expression profile of abiotic stress-responsive genes. Previous studies on the transcriptome in allopolyploids used a limited number of artificially produced lines. Therefore, further studies, including detailed systematic analyses, will be needed to explain the molecular basis of modifications of D-genome variation patterns following allopolyploidization.

## Figures and Tables

**Table 1.** Origin and identity of *Ae. tauschii* accessions used in this study.

**Origin**	**Accession number**
Afghanistan	KU-2059^a^, PI476874^b^, KU-2027
Armenia	KU-2811^a^, KU-2816
Azerbaijan	IG47188, IG47193
China	PI499262^b^, AT47^c^, AT76^c^
Dagestan	KU-20-1, IG120866
Georgia	AE454^c^, KU-2829A^c^
Kazakhstan	AE1090
Kyrgyzstan	IG131606^c^
India	IG48042^c^
Iran	KU-2076^b^, KU-2078^a^, KU-2093^b^, KU-2126^b^, KU-2160^c^, KU-2069, KU-2088
Pakistan	IG46663^a^
Syria	IG46623, IG47259
Tajikistan	IG48554^a^
Turkmenistan	IG48518, IG126387

KU: Plant Germ-Plasm Institute, Faculty of Agriculture, Kyoto University, Japan.

PI: National Small Grains Research Facility, USDA-ARS, USA.

IG: International Centre for Agricultural Research in the Dry Areas (ICARDA), Syria.

AE: Institut für Pflanzengenetik und Kulturpflanzenforschung (IPK), Germany.

AT: Faculty of Agriculture, Okayama University, Japan.

a: Parental accessions for synthetic hexaploids produced in this study.

b: Parental accessions for synthetic hexaploids reported in Takumi *et al*. [50].

c: Parental accessions for synthetic hexaploids from triploids in Matsuoka *et al*. [33].

## References

[1] Koornneef M Alonso-Blanco C Vreugdenhil D Naturally occurring genetic variation in *Arabidopsis thaliana* Annu. Rev. Plant Biol 2004 55 141 172 15377217 10.1146/annurev.arplant.55.031903.141605

[2] Alonso-Blanco C Gomez-Mena C Llorente F Koornneef M Salinas J Martinez-Zapater JM Genetic and molecular analyses of natural variation indicate *CBF2* as a candidate gene for underlying a freezing tolerance quantitative trait locus in *Arabidopsis* Plant Physiol 2005 139 1304 1312 16244146 10.1104/pp.105.068510 PMC1283767

[3] Hannah MA Wise D Freund S Fiehn O Heyer AG Hincha DK Natural genetic variation of freezing tolerance in Arabidopsis Plant Physiol 2006 142 98 112 16844837 10.1104/pp.106.081141 PMC1557609

[4] Bouchabke O Chang F Simon M Voisin R Pelletier G Durand-Tardif M Natural variation in *Arabidopsis thaliana* as a tool for highlighting differential drought responses PLoS ONE 2008 3 e1705doi:10.1371/journal.pone.0001705. 18301780 10.1371/journal.pone.0001705 PMC2246160

[5] Thomashow MF Plant cold acclimation: Freezing tolerance genes and regulatory mechanisms Annu. Rev. Plant Physiol. Plant Mol. Biol 1999 50 571 599 15012220 10.1146/annurev.arplant.50.1.571

[6] Yamaguchi-Shinozaki K Shinozaki K Transcriptional regulatory networks in cellular responses and tolerance to dehydration and cold stresses Annu. Rev. Plant Biol 2006 57 781 803 16669782 10.1146/annurev.arplant.57.032905.105444

[7] Leung J Giraudat J Abscisic acid signal transduction Annu. Rev. Plant Physiol. Plant Mol Biol 1998 49 199 222 15012233 10.1146/annurev.arplant.49.1.199

[8] Shinozaki K Yamaguchi-Shinozaki K Seki M Regulatory network of gene expression in the drought and cold responses Curr. Opin. Plant Biol 2003 6 410 417 12972040 10.1016/s1369-5266(03)00092-x

[9] Xiong L Zhu JK Regulation of abscisic acid biosynthesis Plant Physiol 2003 133 29 36 12970472 10.1104/pp.103.025395 PMC523868

[10] Palva ET Gene expression under low temperature stress Stress Induced Gene Expression in Plants Basra AS Harwood Academic Publishers New York, NY, USA 1994 103 130

[11] Lång V Palva ET The expression of a *rab*-related gene, *rab18*, is induced by abscisic acid during the cold acclimation process of *Arabidopsis thaliana* (L.) Heynh Plant Mol. Biol 1992 20 951 962 1463831 10.1007/BF00027165

[12] Baker SS Wilhelm KS Thomashow MF The 5′-region of *Arabidopsis thaliana cor15a* has *cis*-acting elements that confer cold-, drought- and ABA-regulated gene expression Plant Mol. Biol 1994 24 701 713 8193295 10.1007/BF00029852

[13] Yamaguchi-Shinozaki K Shinozaki K A novel *cis*-acting element in an *Arabidopsis* gene is involved in responsiveness to drought, low-temperature, or high salt stress Plant Cell 1994 6 251 264 8148648 10.1105/tpc.6.2.251 PMC160431

[14] Choi H Hong J Ha J Kang J Kim SY ABFs, a family of ABA-responsive element binding factors J. Biol. Chem 2000 275 1723 1730 10636868 10.1074/jbc.275.3.1723

[15] Uno Y Furihata T Abe H Yoshida R Shinozaki K Yamaguchi-Shinozaki K *Arabidopsis* basic leucine zipper transcription factors involved in an abscisic acid-dependent signal transduction pathway under drought and high-salinity conditions Proc. Natl. Acad. Sci. USA 2000 97 11632 11637 11005831 10.1073/pnas.190309197 PMC17252

[16] Kim SY The role of ABF family bZIP class transcription factors in stress response Physiol. Plant 2006 126 519 527

[17] Tsuda K Tsvetanov S Takumi S Mori N Atanassov A Nakamura C New members of a cold-responsive *Lea*/*Rab*-related *Cor* gene family from common wheat (*Triticum aestivum* L.) Genes Genet. Syst 2000 75 179 188 11126566 10.1266/ggs.75.179

[18] Ohno R Takumi S Nakamura C Kinetics of transcript and protein accumulation of a low-molecular-weight wheat LEA D-11 dehydrin in response to low temperature J. Plant Physiol 2003 160 193 200 12685035 10.1078/0176-1617-00925

[19] Kobayashi F Takumi S Egawa C Ishibashi M Nakamura C Expression patterns of low temperature responsive genes in a dominant ABA-less-sensitive mutant line of common wheat Physiol. Plant 2006 127 612 623

[20] Kobayashi F Ishibashi M Takumi S Transcriptional activation of *Cor*/*Lea* genes and increase in abiotic stress tolerance through expression of a wheat *DREB2* homolog in transgenic tobacco Transgenic Res 2008 7 755 767 18034365 10.1007/s11248-007-9158-z

[21] Jaglo KR Kleff S Amundsen KL Zhang X Haake V Zhang JZ Deits T Thomashow MF Components of the *Arabidopsis* C-repeat/dehydration responsive element binding factor cold-responsive pathway are conserved in *Brassica napus* and other plant species Plant Physiol 2001 127 910 917 11706173 PMC129262

[22] Shen YG Zhang WK He SJ Zhang JS Liu Q Chen SY An EREBP/AP2-type protein in *Triticum aestivum* was a DRE-binding transcription factor induced by cold, dehydration and ABA stress Theor. Appl. Genet 2003 106 923 300 12647068 10.1007/s00122-002-1131-x

[23] Kume S Kobayashi F Ishibashi M Ohno R Nakamura C Takumi S Differential and coordinated expression of *Cbf* and *Cor*/*Lea* genes during long-term cold acclimation in two wheat cultivars showing distinct levels of freezing tolerance Genes Genet. Syst 2005 80 185 197 16172531 10.1266/ggs.80.185

[24] Takumi S Shimamura C Kobayashi F Increased freezing tolerance through up-regulation of downstream genes via the wheat *CBF* gene in transgenic tobacco Plant Physiol Biochem 2008 46 205 211 18061465 10.1016/j.plaphy.2007.10.019

[25] Kobayashi F Maeta E Terashima A Kawaura K Ogihara Y Takumi S Development of abiotic stress tolerance via bZIP-type transcription factor LIP19 in common wheat J. Exp. Bot 2008 59 891 905 18326864 10.1093/jxb/ern014

[26] Kobayashi F Maeta E Terashima A Takumi S Positive role of a wheat *HvABI5* ortholog in abiotic stress response of seedlings Physiol. Plant 2008 134 74 86 18433415 10.1111/j.1399-3054.2008.01107.x

[27] Eig A Monographisch-kritische Übersicht der Gatteung *Aegilops* Repertorium Specierum Novarum Rgni Vegetabilis. Beihefte 1929 55 1 228

[28] Van Slageren MW Wild Wheats: A Monograph of Aegilops L and Amblyopyrum (Jaub & Spach) Eig (Poaceae) Wageningen Agricultural University Press Wageningen, The Netherlands 1994 326 344

[29] Kihara H Discovery of the DD-analyser, one of the ancestors of *Triticum vulgare* (in Japanese) Agric. Hortic 1944 19 889 890

[30] McFadden ES Sears ER The artificial synthesis of *Triticum spelta* Rec. Genet. Soc. Am 1994 13 26 27

[31] Dudnikov AJ Goncharov NP Allozyme variation in *Aegilops squarrosa* Hereditas 1993 119 117 122

[32] Dvorak J Luo MC Yang ZL Zhang HB The structure of the *Aegilops tauschii* genepool and the evolution of hexaploid wheat Theor. Appl. Genet 1998 97 657 670

[33] Matsuoka Y Takumi S Kawahara T Natural variation for fertile triploid F_1_ formation in allohexaploid wheat speciation Theor. Appl. Genet 2007 115 509 518 17639301 10.1007/s00122-007-0584-3

[34] Matsuoka Y Takumi S Kawahara T Flowering time diversification and dispersal in central Eurasian wild wheat *Aegilops tauschii* Coss.: genealogical and ecological framework PLoS ONE 2008 3 e3138doi:10.1371/journal.pone.0003138. 18769547 10.1371/journal.pone.0003138 PMC2519791

[35] Matsuoka Y Nishioka E Kawahara T Takumi S Genealogical analysis of subspecies divergence and spikelet-shape diversification in central Eurasian wild wheat *Aegilops tauschii* Coss Plant Syst. Evol 2009 279 233 244

[36] Dudnikov AJ Kawahara T *Aegilops tauschii*: genetic variation in Iran Genet. Resour. Crop Evol 2006 53 579 586

[37] Feldman M Origin of cultivated wheat The World Wheat Book: A History of Wheat Breeding Bonjean AP Angus WJ Lavoisier Publishing Paris, France 2001 3 53

[38] Trethowan RM Mujeeb-Kazi A Novel germplasm resources for improving environmental stress tolerance of hexaploid wheat Crop Sci 2008 48 1255 1265

[39] Kihara H Lilienfeld F A new-synthesized 6x-wheat Hereditas 1949 Suppl 307 319

[40] Matsuoka Y Nasuda S Durum wheat as a candidate for the unknown female progenitor of bread wheat: an empirical study with a highly fertile F_1_ hybrid with *Aegilops tauschii* Coss Theor. Appl. Genet 2004 109 1710 1717 15448900 10.1007/s00122-004-1806-6

[41] Nishikawa K Hybrid lethality in crosses between emmer wheats and *Aegilops squarrosa*. I. Vitality of F_1_ hybrids between emmer wheats and *Ae. squarrosa* var. *typica* Seiken Ziho 1960 11 21 28

[42] Nishikawa K Hybrid lethality in crosses between emmer wheats and *Aegilops squarrosa*. II. Synthetized 6x wheats employed as test varieties Jpn. J. Genet 1962 37 227 236

[43] Kobayashi F Takumi S Nakata M Ohno R Nakamura T Nakamura C Comparative study of the expression profiles of the *Cor*/*Lea* gene family in two wheat cultivars with contrasting levels of freezing tolerance Physiol. Plant 2004 120 585 594 15032820 10.1111/j.0031-9317.2004.0293.x

[44] McKhann HI Gery C Bérard A Léveque S Zuther E Hincha DK Mita SD Brunel D Téoulé E Natural variation in CBF gene sequences, gene expression and freezing tolerance in the Versailles core collection of *Arabidopsis thaliana* BMC Plant Biol 2008 8 105doi:10.1186/1471-2229-8-105. 18922165 10.1186/1471-2229-8-105 PMC2579297

[45] Comai L Genetic and epigenetic interactions in allopolyploid plant Plant Mol. Biol 2000 43 387 399 10999418 10.1023/a:1006480722854

[46] Kashkush K Feldman M Levy AA Gene loss, silencing and activation in a newly synthesized wheat allotetraploid Genetics 2002 160 1651 1659 11973318 10.1093/genetics/160.4.1651 PMC1462064

[47] Madlung A Masuelli RW Watson B Reynolds SH Davison J Comai L Remodelling of DNA methylation and phenotypic and transcriptional changes in synthetic Arabidopsis allotetraploids Plant Physiol 2002 129 733 746 12068115 10.1104/pp.003095 PMC161697

[48] Wang J Tian L Madlung A Lee HS Chen M Lee JJ Watson B Kagochi T Comai L Chen ZJ Stochastic and epigenetic changes of gene expression in Arabidopsis polyploids Genetics 2004 167 1961 1973 15342533 10.1534/genetics.104.027896 PMC1471021

[49] Pumphrey M Bai J Laudencia-Chingcuanco D Anderson O Gill B Nonadditive expression of homoeologous genes is established upon polyploidization in hexaploid wheat Genetics 2009 181 1147 1157 19104075 10.1534/genetics.108.096941 PMC2651049

[50] Terashima A Takumi S Allopolyploidization reduces alternative splicing efficiency for transcripts of the wheat *DREB2* homolog, *WDREB2* Genome 2009 52 100 105 19132077 10.1139/G08-101

[51] Takumi S Naka Y Morihiro H Matsuoka Y Expression of morphological and flowering time variation through allopolyploidization: an empirical study with 27 synthetic wheats and their parental *Aegilops tauschii* accessions Plant Breed 2009 12doi:10.1111/j.1439-0523.2009.01630.x.

